# Deepfakes and Democracy (Theory): How Synthetic Audio-Visual Media for Disinformation and Hate Speech Threaten Core Democratic Functions

**DOI:** 10.1007/s44206-022-00010-6

**Published:** 2022-09-08

**Authors:** Maria Pawelec

**Affiliations:** Department of Society, Culture and Technological Change, International Center for Ethics in the Sciences and Humanities (IZEW), University of Tübingen, Wilhelmstrasse 19, 72074 Tübingen, Germany

**Keywords:** Deepfakes, Synthetic media, Democracy theory, Technology ethics, Media ethics

## Abstract

Observers fear that deepfakes will shake the very foundations of democracy. Notwithstanding, in-depth scholarly analyses of deepfakes’ political impact are rare, and do not consider theories of democracy. This contribution helps close this research gap, drawing on Warren’s problem-oriented democracy theory, as well as theories of deliberative democracy and contributions on the role of trust in democracies. I identify three core functions of democratic systems and their normative foundations, namely empowered inclusion, collective agenda and will formation (supported by deliberation), and collective decision-making. Based on a literature and media analysis, I systematize different types of deepfakes serving either disinformation or hate speech and outline how they weaken core democratic functions and norms: Deepfakes impede citizens’ empowered inclusion in debates and decisions that affect them, e.g. by hampering efforts to hold political representatives accountable or further marginalizing certain societal groups such as women or ethnic minorities. Deepfakes also undermine collective agenda and will formation by threatening the epistemic quality of deliberation as well as citizens’ mutual empathy and respect. This culminates in a decreased legitimacy of collective decisions taken, which is additionally threatened by pervasive (but mostly speculative) fears of deepfake election manipulation. My analysis has implications for (future) governance efforts addressing deepfakes. Such efforts are increasing, e.g. on the part of social media platforms, but also (supra-)national regulatory bodies.

In 2020, deepfakes, i.e. synthetic audio-visual media of human faces, bodies, or voices, are often created using artificial intelligence (AI),^1^ “went mainstream” (Hao & Heaven, 2020). Besides fake porn, mundane and prosocial uses increased significantly—as did the technology’s political applications. The public debate about deepfakes centres mainly on this (malicious) political potential, as a recent discourse analysis shows (Gosse & Burkell, 2020). Policy analysts fear deepfakes’ “challenge to truth in politics” (Galston, 2020), human rights organizations believe that the technology may be exploited by authoritarian regimes (Gregory, 2021), and journalists even see democracy endangered in general (e.g. Frum, 2020; Parkin, 2019; Thomas, 2020).

The threat posed by deepfakes is not entirely new. Media manipulation with both harmful and benign intent is as old as media itself. E.g. convincing image manipulation has a long trajectory for art, but also non-consensual pornography (Burkell & Gosse, 2019) and disinformation, i.e. “false, inaccurate, or mis-leading information designed, presented and promoted to intentionally cause public harm or for profit” (HLEG, 2018: 10).^2^ In this sense, deepfakes are just a new means of media manipulation. However, the technology is developing in an information environment already challenged by the way (dis)information is shared and spread via social media (Schick, 2020). Deepfakes’ characteristics such as their increasing quality and persuasiveness also amplify challenges posed by manipulated media: In the past, it was difficult to convincingly manipulate audio and video material, increasing trust in such media. Also, audio and video “appeal[s] at a visceral level more than any text or picture ever will” and is thus often perceived as particularly credible (Kwok & Koh, 2020: 1; see also Kietzmann et al., 2020: 136). AI has now greatly simplified and improved the manipulation and synthesis of audio and video (as well as images). This is beneficial, e.g., for commercial or artistic uses of deepfake technology, but has also increased their deceptive potential. Accordingly, in a rare experimental study on deepfakes’ effect on political attitudes, less than fifteen percent of participants doubted the authenticity of a deepfake video of a politician shown to them (Dobber et al., 2020: 78). Deepfakes’ increasing quality also means that many can no longer be uncovered without technical support, aggravating harmful deepfakes’ governance. When perfected, synthetic media will no longer be based on discernible authentic raw material at all (Diresta, 2020), making them even harder to detect—and easy to generate once the initial model is completed. Besides their realism, deepfakes’ accessibility raises concerns: Unlike earlier forms of sophisticated media manipulation, deepfake generation is increasingly commercialized and accessible even to laypersons (Ajder et al., 2019: 5). Accessibility has caused a massive surge in deepfakes and new applications. This includes benign uses, but, e.g., also deepfake revenge porn, posing new ethical and societal challenges. Deepfakes thus greatly exacerbate existing (political) concerns associated with manipulated media, and technological progress suggests this trend will only continue.

It is thus vital to assess deepfakes’ societal and political impact in detail. However, few scholars and policy analysts have done so to date. Social sciences and humanities research on deepfakes is rare overall and does not relate deepfakes’ impact to core democratic norms. Greater theoretical grounding and conceptual clarity are needed to help specify and evaluate the harm (and potential) of current and future deepfake applications. This will also provide a sounder basis for societal and political responses to deepfakes. As Tenove (2020: 520) argues concerning disinformation, varying understandings of threats posed to democracy “can be used to design and justify quite different policies”. These policies in turn (dis)advantage different actors and may themselves threaten core democratic norms. It is thus crucial to specify the democratic goods threatened by deepfakes to craft adequate policy responses and protect democracy.

This contribution attempts to answer the following research questions: (How) Do deepfakes threaten democracy? Which underlying normative goods or values do they affect?^3^ Thereby, deepfakes’ impact depends heavily on their context of use and the intentions behind it.^4^ Many deepfakes, e.g. in the areas of political activism, art, and education, may even strengthen democratic debate and institutions (see Pawelec, 2022). However, this paper focuses explicitly on deepfakes’ *harmful* potential for democracy as this lies at the centre of debate about deepfakes but nonetheless remains understudied.

To answer the paper’s research questions, I draw on systemic democracy theory that specifies the functions or normative goods a system must realize to be considered democratic. I integrate Warren’s (2017) “problem-based approach” to democracy theory and deliberative democracy theory, in particular the systemic approach by Mansbridge et al. (2012). I identify three core democratic functions and their normative foundations: democratic systems must enable empowered inclusion, collective agenda and will formation, and collective decision-making.^5^ Deliberation, in this view, is a social practice that mainly supports collective agenda and will formation and entails certain epistemic and ethical benefits. Within this framework, trust is also essential to ensure citizens’ participation in deliberation (allowing for empowered inclusion and the shaping of collective agendas) and to enable efficient political decision-making.

Based on this theoretical framework, I assess claims about different deepfakes’ harmful political impact. I augment this with a technology ethical analysis of recent deepfake use cases. Thereby, my contribution assesses a large body of literature on deepfakes’ impact, which is dispersed, often cursory, and lacking an ethical or political science focus. Empirically, the analysis is based on over 300 academic contributions, media reports, and internet publications. It is structured according to an inductive systematization of six different deepfake applications and types of impact in the political context: Three deepfake uses spread disinformation, serving differing political goals (deepfakes for election manipulation, targeted attacks on political opponents, and foreign interference). The associated trust decay permits doubt or a denial of inconvenient facts (the “liar’s dividend”) and weakens news media. Additionally, I consider pornographic and other demeaning deepfakes without obvious political agenda.

I argue that such pornographic deepfakes are politically relevant as they constitute hate speech. My analysis shows that they mainly threaten the core democratic function and norm of empowered inclusion. It discourages certain societal groups, in particular women, from participating in the public sphere—aggravating existing discrimination.

Besides, both domestic and foreign actors create deepfakes for the purpose of disinformation, often within the context of elections, but increasingly also to target, e.g., critical journalists and dissidents. Such deepfakes undermine trust in democratic societies. They hamper inclusion by discounting citizens from relevant political debates or candidates from elections, marginalizing certain societal groups, or preventing citizens from voting. They also threaten *empowered* inclusion when they skew deliberation, especially during elections, to a degree that prevents citizens from making rational voting decisions and holding representatives accountable. Besides, deepfake disinformation threatens collective agenda and will formation, mainly through the infusion of falsehoods into democratic deliberation and thus the erosion of epistemic quality. Besides, deepfakes’ mere existence enables actors to strategically deflect blame and deny uncomfortable facts. Polarizing deepfakes also undermine mutual respect, the willingness to consider opposing opinions and arguments, and thus deliberation. Taken together, these consequences decrease the legitimacy of collective decisions. Additionally, the fear of deepfakes during elections alone is sufficient to undermine trust in this core democratic practice, which is vital for decision-making.

I conclude by identifying limitations of my contribution and fruitful avenues for future research, including a stronger focus on deepfakes’ positive political potential. I also identify key implications of my analysis for (future) governance efforts addressing the challenges deepfakes pose to democracy.

## The Debate About Deepfakes and Democracy

Despite the media and politics’ overwhelming emphasis on deepfakes’ potential for political abuse, in-depth analyses thereof are rare. They also mostly do not highlight the values at stake and are not explicitly based on democracy theory. According to a recent systematic review of deepfake research published 2018–2020, most studies either stem from computer sciences or law and focus either on deepfake detection or regulation. Humanities or social sciences analyses are rare (Godulla et al., 2021: 82).^6^ Philosophers have recently explored deepfakes’ relation to authenticity (Floridi, 2018), the inherent moral wrong of deepfakes (Ruiter, 2021), and the “epistemic threat” they pose (Fallis, 2020). However, while the latter sheds light on how deepfakes affect viewers, it is based on theories of information carrying rather than democracy theory, and only cursorily mentions the “epistemic harms” incurred.

The above-mentioned review article also shows that most deepfake research focuses on deepfakes’ risks, including challenges to journalism and trust in (social) media (Godulla et al., 2021: 81, 85). However, concerning threats to “political campaigns […], public opinion and […] trust in institutions”, the review identifies only one contribution, i.e. (Chesney & Citron, 2019). Consequently, “the context of political news and election campaigns” is seen as an important avenue for future research (ibid.: 91).

Chesney and Citron (2019: 1758) provide the “first comprehensive survey” of deepfake-induced harms, focusing on the USA. Their seminal contribution coins the term “liar’s dividend” and provides insights, e.g., into the gendered dimension of deepfake porn (ibid.: 1773). It also refers to values such as autonomy and privacy and highlights the dependence of a functioning democratic discourse on “shared facts and truths” (ibid.: 1770, 1777). However, it has a legal focus and is not based on an explicit ethical framework. Consequently, it does not explicate the democratic norms deepfakes threaten.^7^

Similarly, in a popular science monography on deepfakes, policy consultant Schick (2020) analyses political use cases, including the silencing of an Indian journalist through deepfake porn, and deepfake-fuelled destabilization in Gabon. Schick also predicts new dangers such as deepfakes undermining trust in audio-visual proof of human rights violations and amplifying the liar’s dividend. She offers a broad account of deepfakes’ political impact but often entangles this with a more general analysis of disinformation. Her account is also not based on an explicit theoretical framework or democracy theory.

Reports, e.g. by think tanks and start-ups, outline deepfakes’ political impact, again without explicit reference to democratic norms (e.g. Smith & Mansted, 2020; Ajder et al., 2019). An in-depth report by the German Konrad-Adenauer-Stiftung and the Counter Extremism Project on deepfakes’ threat to democracy outlines several cases of deepfakes “disrupt[ing] democratic elections and sow[ing] civil unrest” (Farid & Schindler, 2020: 24). However, the actual analysis of political uses only covers three pages and does not reference democracy theory or core democratic norms (ibid.: 24–26). Instead, the report aims mainly at stimulating political and societal debate in Germany on countering deepfakes. Similarly, a study prepared for the European Parliament in 2021 covers technical, societal and regulatory aspects of deepfakes (van Huijstee et al., 2021). The report discusses potential “damage[s] to democracy” as one of several “risk[s] of societal harms” caused by deepfakes, again without referencing democracy theory (ibid.: 31-34). Its main focus lies on assessing the existing regulatory landscape and proposing policy options for regulation on a European level.^8^

By contrast, Diakopoulos and Johnson (2019) seek to outline “the ethical implications of deepfakes in the context of elections” using an anticipatory approach. However, they describe their scenario development in detail but base their ethical reflection only on methodological literature on the ethics of emerging technologies, rather than an explicit ethical framework (ibid: 5). They also focus solely on the (then-upcoming) 2020 US presidential elections.

Jaiman (2020) of the Microsoft “Defending Democracy Program” broadly purports to debate “the ethics of deepfakes” in a think tank anthology. However, he only dedicates one paragraph to analysing deepfakes’ impact on democracy, repeatedly stating that certain deepfakes are “unethical” or “immoral”, without specifying the normative goods at stake (ibid.: 77).

Discussing informational warfare and political subversion, Paterson and Hanley (2020: 448–449) purport that deepfakes will aggravate the success of political warfare campaigns and impede their detection. However, they only give one example of such a deepfake (an edited video of the US House of Representatives Speaker Nancy Pelosi, ibid: 448), and this video is, in fact, not a deepfake, but a “cheapfake”, i.e. a video edited with less sophisticated means than deepfake technology.

In a recent contribution, Etienne (2021) argues that deepfakes do not threaten but may rather increase online trust (I engage with this argument in footnote 20). While trust is crucial for democracies, Etienne does not connect his argument with democracy theory.

More political science, philosophy, and applied ethics research on deepfakes is thus needed. Specifically, in-depth analyses of deepfakes’ political impact, linked to theories of democracy, are lacking^9^—although its disruptive potential for democracy is arguably the greatest fear associated with deepfake technology. The present contribution addresses this research gap.

## Theoretical Framework

(Political) Philosophers and other thinkers have long engaged with justifications for democracy (and related political ideas) and its normative foundations. To enable a stringent analysis of the core democratic norms threatened by deepfakes, I will not trace and consider their numerous and diverse theories. Instead, my theoretical framework is inspired by a recent contribution by Tenove (2020) on disinformation and democracy since most malicious political uses of deepfakes serve disinformation as instances of media manipulation by new means. Tenove (2020) seeks to bring more conceptual and theoretical clarity into the debate about disinformation’s impact on democracy, which is lacking despite the topic’s political pervasiveness following the 2016 US elections. He builds on systemic approaches to democratic theory that specify which normative goods political systems need to foster to be considered democratic without prioritizing individual goods (ibid.: 521). In particular, Tenove draws on Warren’s (2017) problem-based democracy theory and additionally considers theories of deliberative democracy.

While I draw on a similar theoretical framework, the core democratic goods I identify differ from Tenove’s.^10^ I refer more closely to Warren’s original theory and, e.g., place greater emphasis on the value of empowered inclusion. I also more seamlessly integrate deliberative democratic theory into my theoretical framework and highlight the role of trust for democracies.^11^ This is particularly important, as fears prevail that deepfakes will undermine trust in democratic societies and a shared belief in and agreement on certain facts.

Warren (2017) asks which problems a political system must solve to be considered democratic, i.e. which “democratic functions” it must fulfil (ibid.: 41–43). The mere existence of specific institutions or informal arrangements is not sufficient for a system to be considered democratic. Rather, certain practices and (in)formal institutions such as deliberation or voting with their specific strengths and weaknesses serve or impede the realization of the different democratic functions (ibid.: 39).

Building on what he regards as a degree of consensus among democracy theorists, Warren suggests three such core democratic functions and specifies their normative content: systems must enable empowered inclusion, collective agenda and will formation, and collective decision-making (ibid.). *Empowered inclusion* is based on the normative idea that those (potentially) affected by a collective decision should participate in its formation (ibid.: 44). Besides this normative entitlement, democracies also distribute powers that allow the affected to claim and enforce such participation, e.g. through voting, representation, or association. Political equality in terms of rights and protections is a “core democratic value” here (ibid.). Democracies also need to enable *collective agenda and will formation*, i.e. the translation of individuals and groups’ preferences, interests, and values into collective agendas and wills. According to Warren (2017: 44), referring, e.g., to Habermas (1990) and Rawls (2001), this is based on moral and ethical considerations such as the need for mutual recognition, respect, and reciprocity (which in turn enhances deliberation), as well as fairness. Finally, democracies need to empower collectives (not necessarily restricted to nation-states) to take joint decisions on relevant issues (*collective decision-making*; Warren, 2017: 43). This serves performance or output legitimacy (ibid.: 45). Warren (2017: 45ff) then identifies seven practices or social actions that contribute to the three democratic functions, including voting, representation, and deliberation.

Concerning deliberative democracy theory, Warren (2017: 40) argues that it is an important research paradigm but that it overemphasizes the significance of deliberation for democracies and, e.g., neglects voting. Deliberation can but does not necessarily enhance empowered inclusion or collective decision-making. Its main strength lies in communication and thus collective agenda and will formation (ibid.: 48)—an assessment mirrored, e.g., by Habermas (2005: 287) in his elaboration of normative “discourse theory”. In Warren’s view, deliberation as negotiating, exchanging arguments, and bargaining is thus only one of several social practices that further the three core democratic functions. However, besides facilitating collective will formation (and enhancing decision legitimacy), Warren (2017: 48) identifies several other goods furthered by deliberation, in particular “epistemic goods” and “ethical benefits” such as mutual empathy and understanding.

This elaboration serves as a point of reference for me to link Warren’s problem-based approach with deliberative democratic theory. Systemic approaches to deliberative democracy regard how the ideal of deliberation functions within larger collectives and suggest a division of deliberative labour between different arenas and institutions (e.g. Chambers, 2017; Habermas, 2005: 288; Mansbridge et al., 2012: 9).^12^ While there are numerous theories of deliberative democracy, Mansbridge et al. (2012: 11–12) identify three normatively relevant functions of deliberation which are allegedly quite uncontroversial: an epistemic, an ethical, and a democratic function.

Deliberation’s *epistemic* function lies in the formation of political interests, opinions, and decisions through rational discussion and an exchange of ideas grounded in facts, logic, and the mutual consideration of arguments (ibid.: 11). Thus, like Warren, Mansbridge et al. regard epistemic quality as a good that follows from deliberation (which, in turn, facilitates collective agenda and will formation according to Warren). Like Warren, Mansbridge et al. also identify an *ethical* function of deliberation, namely, to foster citizens’ “mutual respect”. This ethical function entails a recognition of citizens as autonomous agents capable of contributing to political discourse. Simultaneously, mutual respect is an intrinsic element of deliberation and a prerequisite for effective communication (ibid.). Lastly, Mansbridge et al. (2012: 12) see deliberation’s *democratic* function in its support of an inclusive and equal political process—akin to Warren’s function of empowered inclusion.

However, according to Warren, deliberation can but does not necessarily enhance empowered inclusion, since it does not per se legitimate the inclusion of certain groups in the political process or empower them. Deliberation in skewed power contexts can even undermine democracy (Warren, 2017: 48). This corresponds to analyses that suggest that deliberation enhances power asymmetries under certain circumstances (e.g. Lupia & Norton, 2017). Interestingly, Mansbridge et al. (2012: 12) support this assessment: rather than specifying how deliberation enhances inclusion, they merely state that inclusion is “what makes deliberative democratic processes democratic”. Both theoretical frameworks thus indicate that (empowered) inclusion is normatively desirable in deliberative processes but that deliberation does not necessarily strengthen it.

Besides, a certain degree of *trust* is a prerequisite for deliberation, as it ensures citizens’ participation and engagement (Parvin, 2015: 417). Trust is also essential for the formation of an informed and shared democratic public (Coleman, 2012: 36) and the efficiency of collective decision-making. Lastly, societal trust is connected to mutual respect, enabling empowered inclusion. Trust is thus crucial for democracy, and this includes both what may be deemed “informational trust”, i.e. trust in what one sees and hears and in shared facts and truths, and “societal trust”, e.g. in fellow citizens, political processes, and institutions.

Based on rational choice theory, informational trust arises as people expect greater benefit from believing information and shared facts than from having to fact-check everything themselves (Etienne, 2021: 556). Societal trust, on the other hand, is defined by Warren (1999: 2) in an anthology on trust and democracy as granting others power over some good and accepting a certain degree of vulnerability towards them to benefit from cooperation.^13^ This conception of trust is also based on rational choice theory (Etienne, 2021: 556). I will extend it here building on a recent contribution on deepfakes and trust, in which Etienne (2021, 557) argues that societal trust also reflects peoples’ wish to build relationships with other people, and their prioritization thereof over other available information in decision-making. Trust, then, is not just a means to an end but an end in itself.

The present paper builds on Warren’s problem-based, normative approach to democratic theory and links this closely with theories of deliberative democracy and contributions on the role of trust for democracy. This constitutes the theoretical framework for my analysis of different uses of deepfake technology and their impact on democracy.

## Methodology

Empirically, this paper is based on an analysis of more than 300 academic contributions, media reports, and internet publications (e.g. by think tanks, start-ups, and civil society) on deepfakes’ ethical and societal implications. Thereby, I include cases from (semi-)authoritarian states, since my focus is not on how deepfakes affect states widely acknowledged as “democracies” but on the core values and norms of democracy. Academic contributions were uncovered by repeatedly searching for the terms “deepfake” and “deep fake” via Google Scholar from January 2020 to May 2021 and then employing a snowball system based on the references. A systematic analysis of Google Alerts for the search terms “deepfake”, “deep fake”, and “synthetic media” (and German equivalents) from June 2020 to April 2021 uncovered the media reports and internet publications.^14^ All reviewed sources were published in English or German and added to a Citavi database on deepfakes comprising over 600 titles.

The analysis relies on very different types of sources, since peer-reviewed academic contributions on the topic are rare and assess only few actual uses of deepfakes. My analysis thus draws heavily on reports, e.g., by civil society organisations or state bodies and the media. This is crucial to paint as comprehensive and evidence-based a picture of deepfakes’ harmful political impact as possible. However, the varying nature of sources may have negative implications for the analysis. To ensure the validity of information, several factors were considered, including the reputation of sources such as civil society organisations or news media, the reception of content (i.e. was information cited again by reliable sources, e.g. in peer-reviewed journals), and triangulation (i.e. did the information given by several sources correspond). To ensure transparency, I have also added a list of all (types of) sources used to assess different deepfake use cases in the paper’s following sections as an appendix to the article (Table 1).^15^

To enable analysis, the uncovered sources were summarized and important quotes transferred into a 48-page working document on deepfakes’ political impact. To structure and assess the material, I then took a problem-based approach. Similar to Tenove’s (2020) assessment of disinformation’s impact on democracy, I structured the available literature on deepfakes’ impact by “toggling between identifying emergent categories in the data and engaging with concepts from democratic theory and media studies” (ibid.: 520)^16^ as well as from the literature on deepfakes’ (potential) impact cited in my literature review.

Since the existing literature on deepfakes’ political impact nearly exclusively focuses on deepfake-based disinformation, this emerged as the first overarching use of deepfakes. A closer analysis revealed three different uses of deepfakes to spread disinformation and two associated effects (the liar’s dividend and a weakening of the media). Additionally, the broader deepfake literature clearly identifies pornography as their main use and discusses its implications primarily for individuals. I argue that such deepfakes are also politically relevant and thus added deepfake hate speech as a second overarching type of deepfake use that may harm democracy.

Within this structure, the actual analysis then proceeded in two steps: First, a literature review was conducted based on the above-mentioned working document. The information available on individual use cases served to detail each specific use of deepfakes, its context, reach, and immediate impact. Thereby, I attempted to give as comprehensive an overview of specific deepfake uses to date as possible, rather than highlighting only particularly spectacular or well-known examples or instances which support a certain argument.^17^ The broader literature on deepfakes was also considered to offer a more in-depth assessment, including of deepfakes’ potential in certain fields. In a second step, the analysis then related deepfakes’ current and future uses to the paper’s theoretical framework.

## Deepfake Disinformation and Democracy

Scholars’ and journalists’ main fear concerning deepfakes and democracy is arguably that deepfakes will erode trust within democratic societies and thus democracy itself. This argument concerns deepfake *disinformation* and is two-fold: Firstly, by creating doubt over “what one sees and hears”, deepfakes undermine the factual basis of deliberation and can contribute to a siloed, “post-fact” society. Deepfakes threaten trust in shared facts and truths and thus contribute to what may be deemed an “informational trust decay”^18^: When democratic discourse can no longer build upon shared facts and truths, deliberation cannot sufficiently serve its epistemic function, i.e. produce epistemic quality.^19^ This skews and impedes the core democratic function of collective agenda and will formation. Chesney and Citron (2019: 1777–1778) highlight this effect in their seminal contribution on deepfakes, stating that deepfakes may enable individuals to live their own personal truths, undermining any shared understanding of empirical facts and thus democratic discourse (see also, e.g., Bovenschulte, 2019: 1 and Diakopoulos & Johnson, 2019: 11 and a respective overview of news media reporting in Gosse & Burkell, 2020: 503). Informational trust decay also hampers rational collective decision-making, as problem-solving becomes stalled and embroiled in unnecessary discussions over factual claims (Chesney & Citron, 2019: 1777).

By extension, deepfakes also threaten trust in fellow citizens, news media, and (other) democratic institutions and processes such as elections. This broader, “societal trust decay” also endangers democracy: A certain degree of (mutual) trust is necessary for the organization of complex societies, since it is impossible for individual citizens to participate in all political processes affecting them (Warren, 1999: 3–4). As shown, it is also a prerequisite for participation in deliberation. Societal trust decay thus diminishes citizens’ capacity and willingness to organize politically and to efficiently solve collective problems, threatening both empowered inclusion and collective decision-making.^20^

Scepticism of political institutions is a long-standing phenomenon (Etienne, 2021: 559). Distrust in facts and strategic efforts to fuel it are also not new or specific to deepfakes but rather a core feature of disinformation (see, e.g., Chambers, 2021: 149). However, as argued above, deepfakes’ characteristics and their proliferation amplify the phenomenon. But is the trust decay caused by deepfakes really triggering an “infocalypse”, i.e. a collapse of the informational ecosystems in democratic societies (Schick, 2020)? Or is deepfakes’ harmful impact on democracies overestimated? This paper contributes to answering these questions by analysing, in a next step, three use cases of deepfakes for disinformation that contribute to such trust decay, namely election manipulation, attacking political opponents, and foreign interference, and how they negatively affect the core functions of democracy. I then assess the so-called liar’s dividend and how deepfakes are contributing to weakening the news media and journalism.^21^

### Election Manipulation

A widespread fear is deepfakes’ use mainly by domestic (and non-state foreign) actors to spread disinformation in the context of elections (see, e.g., Diakopoulos & Johnson, 2019; Chesney & Citron, 2019: 1774–1778).^22^ However, my review of scholarly literature and media reports reveals only few such deepfakes: In 2018, a Flemish socialist party spread doubt about climate change using a crude (and labelled) deepfake video of Trump (Parkin, 2019). Various parties posted manipulated images and videos during the Indian parliamentary elections 2019 (Goel & Frenkel, 2019), but none were deepfakes. In the 2020 US presidential elections, fears of deepfakes were widespread, but largely did not materialize. I found only few (potential) cases here: In March 2020, Trump himself retweeted a fabricated video of Biden pulling grimaces. However, the original post was labelled a deepfake (Frum, 2020), the video is hardly deceptive, and it has been doubted whether it is technically a deepfake (Cole, 2020). In May 2020, Trump tweeted a deepfake of himself superimposed into the film “Independence Day” (Papenfuss, 2020). While technically more clearly a deepfake, its deceptive potential is also doubtful. Similarly, “robocalls” to millions of voters in Michigan in autumn 2020 urged them not to vote or spread disinformation about the elections. However, the caller sounded “robotic” (Romm & Stanley-Becker, 2020), rather than convincingly emulating a human. Also, in October 2020, a fake intelligence document about Biden’s son and his alleged business connections with China surfaced “on the right-wing internet” and was later spread, e.g., by QAnon supporters and Republican politicians to damage Biden’s presidential candidacy (Collins & Zadrozny, 2020). The document’s main author was a fake persona with a deepfake profile picture. Interestingly, this was soon uncovered, but did not impede the document’s dissemination (ibid.). Thus, while the (wider) conspiracy theory surrounding Biden’s son may have impacted the US elections, deepfake-based deception was arguably not decisive.

In my assessment, deepfakes have thus not significantly altered any democratic election to date. A major reason may be that less sophisticated “cheapfakes” or even just the simple misattribution of images and videos are still sufficient to “create [political] turmoil” (Harwell, 2019), as exemplified by cheapfakes of Biden shared in 2020 (Johnson, 2020). Deepfake technology is often still unnecessary and inefficient (Meneses, 2021: 7).^23^

To date, the impact of election-related deepfakes on democracy thus remains mainly speculative. They could undermine inclusion in several ways. Firstly, deepfakes may exclude citizens from debates that concern them or specific candidates if they “drown out” their voices from political fora (Tenove, 2020: 529). More directly, deepfakes that spread disinformation (or fear) about election procedures, and demeaning (e.g. pornographic) deepfakes that blackmail voters into not voting, undermine the practice of voting, which only “functions as empowerment” if it is universal (Warren, 2017: 49). Thirdly, deepfakes may challenge candidates’ fair chances in an election when used for “false claims, conspiracy theories, chauvinistic language, and imagery that stokes moral revulsion toward electoral candidates and public officials” (Tenove, 2020: 528–529). *Empowered* inclusion is threatened when deepfakes skew deliberation to a degree that prevents citizens from holding representatives accountable for political decisions and potential misdemeanour by voting for rivals (Warren, 2017: 48).

Election-related deepfakes also threaten collective agenda and will formation: Disinformation about political candidates and programmes undermines deliberation’s epistemic quality. Respective deepfakes contribute to an environment in which the public no longer trusts what it sees and hears. This hampers or even prevents rational discussions and negotiation within a public sphere which ideally considers and reacts to real-life societal problems (Habermas, 2005: 290). Also, election-related deepfakes aim to increase polarization and decrease mutual respect, which in turn impedes deliberation. Since election-related deepfakes undermine universal empowered inclusion and deliberation’s sound epistemic basis, collective decision-making is also impeded.

Arguably, election-related deepfakes have not had this detrimental effect on democracy yet—although it may well materialize in the (near) future, considering deepfakes’ growing sophistication and (cost and resource) efficiency. Importantly, however, deepfakes have caused great concern among academia, journalists, political actors, and, increasingly, the public. I argue that this fear itself—rather than deepfakes’ actual use in elections—currently constitutes electoral deepfakes’ greatest threat to democracy: It undermines citizens’ and other political stakeholders’ trust in the fairness and integrity of elections (see Diakopoulos & Johnson, 2019: 12). Thus, it fuels societal trust decay. This reduces trust in elected representatives and the legitimacy of collective decisions (see also Habermas, 2005: 288).

### Attacking Political Opponents and Suppressing the Opposition

Deepfakes can also attack political opponents outside of elections. Targets could include public officials, judges, soldiers, agencies, civil society, religious organisations (Chesney & Citron, 2019: 1776, 1779), journalists, dissidents, or activists. Deepfakes fabricating falsehoods could prevent people from engaging, e.g., in protests, by spreading false organisational information or falsehoods about the political issue at stake. Deepfakes could also cause reputational damage, augment existing societal cleavages or even incite intra- or interstate violence (see, e.g., ibid.: 1757, 1776; Bovenschulte, 2019: 3). Besides, intimidating or humiliating targeted deepfakes could “silence” opponents.

A case in point occurred in July 2020, when a pro-Palestinian activist and her husband were accused in a US Jewish newspaper of being “known terrorist sympathizers”. The article’s author was a fake persona using a deepfake profile picture, who had deceived the newspaper (Satter, 2020). One month later, testimonials by fake leftists (with deepfake profile pictures) who had allegedly converted to supporters of Israeli Prime Minister Benjamin Netanyahu appeared on the Facebook page “Zionist spring” and were shared in far-right circles with little regard for their fake nature. The campaign aimed to weaken growing anti-Netanyahu sentiment and protests (Benzaquen, 2020). In February 2021, Facebook also removed 530 Instagram accounts (partially with deepfake profile pictures) originating in Russia aimed at suppressing domestic pro-Navalny protests (Facebook, 2021b).

Concerning silencing through deepfakes, a prominent case is that of Indian journalist Rana Ayyub, who critically reported on the ruling party BJP in 2018 and was subsequently targeted by a deepfake porn video released together with personal data (doxing). The deepfake went viral and even entailed death threats; Ayyub suffered from anxiety attacks and heart palpitations. She has subsequently reported censoring herself (Schick in Jankowicz, 2021; WITNESS, 2020a). Here, deepfake pornography was used to silence a critical voice, blurring the line to politics (and disinformation).^24^

More generally, journalists and human rights organizations increasingly fear targeted deepfakes within tense political climates characterized by little media freedom and literacy, where deepfakes will allegedly be devastating and contribute to the 'shrinking space' e.g., of dissidents and journalists with few resources to debunk them (Boundaoui; Rajagopalan in WITNESS, 2020a). Such environments are not democracies, but pro-democratic actors are targeted. To date, I could only find one (potential) example thereof (while respective “cheapfakes” abound): In March 2021, a (seeming) confession by a Myanmarese minister to have bribed Aung San Suu Kyi was aired on a military-owned TV channel. Many viewers suspected that it was a face-swap deepfake (KrASIA, 2021).^25^ The case (potentially) confirms worries that deepfakes will be used for forced “confessions” (Rajagopolan in WITNESS, 2020a for the Chinese context).

Targeted political deepfakes undermine *empowered* inclusion since their victims (e.g. journalists and dissidents) often serve critical balancing functions, publicizing authorities’ misdemeanour. Their silencing impedes accountable representation. Disinformation campaigns using deepfake profile pictures are also increasingly used to suppress anti-government protests, again undermining citizens’ empowerment. Targeted deepfakes also weaken collective agenda and will formation through an erosion of epistemic quality: Either critical voices or facts are unduly omitted from public deliberation (or at least questioned), or deliberation is infused with disinformation, e.g., through forced confessions. Targeted deepfakes also polarize, undermining citizens’ mutual respect and willingness to engage with opposing ideas and arguments. The combined lack of epistemic quality and mutual respect, and thus deliberative quality, again undermines the legitimacy of collective decision-making.

The dangers deepfakes pose for politically active individuals and organisations are not specific to the technology. Many of the above-mentioned attacks may have occurred even without deepfakes (and some did, as Russian efforts to suppress the opposition using fake Instagram profiles not based on deepfakes show). In tense political climates or societies with low media literacy, it is often sufficient to simply, e.g., show footage taken out of the context to discredit individuals or institutions (Rajagopolan in WITNESS, 2020a). However, as discussed in the introduction, deepfakes are a particularly sophisticated, convincing, and increasingly accessible technology that may also take effect in less challenging political climates and fool institutions (such as news media) and publics with higher levels of media literacy.

### Foreign Interference

Deepfakes can also serve foreign interference, i.e. efforts by “authoritarian state and non-state actors […] to destabilise their democratic counterparts” (Bentzen, 2020: 1). Identifying respective cases is challenging, since it is typically hard to trace deepfakes’ origins. My categorization here relies on judgments by security agencies, social media analysts, and platforms.

In my assessment, deepfakes have only recently served foreign interference and were exclusively used to create deepfake profile pictures for fake social media accounts up until 2022^26^: In September 2020, analytics firm Graphika and Facebook blocked “Operation Naval Gazing”, a Chinese network of such accounts on Facebook and Instagram that posted content on geopolitical issues such as US-Chinese relations and the South China Sea conflict (Bastian, 2020). Another pro-Chinese influence operation, “Spamouflage Dragon”, targeted US citizens in the 2020 elections with pro-Biden messages (Stone, 2020a). In 2020, the US Federal Bureau of Investigation (FBI) also alerted Facebook to a campaign by the Russian “Internet Research Agency” (IRA) using deepfake profiles to amplify societal divisions in the USA with both pro-Biden and pro-Trump content (Vavra, 2021) and conspiracist content surrounding COVID-19 and QAnon (Stone, 2020b). A related Russian operation running a far-right “news” website and accounts on Gab and Parler also targeted the American public with pro-Trump messaging and relied on fake editorial personas (Graphika, 2020). These Russian operations’ goal was to “push […] users toward both ends of the political spectrum with divisive and hyper-partisan content” (ibid.).

In the context of the ongoing war in Ukraine, a (allegedly) foreign-made deepfake *video *materialized: In March 2022, a Ukrainian news agency’s website was hacked to publish a deepfake video of Ukrainian president Zelensky urging Ukrainians to surrender.^27^ The video then spread on social media but never went viral due to its low visual quality and other indications of fakery, including fake Zelensky’s Russian accent and robotic voice (Bastian, 2022). The political context and accent indicate that the deepfake was part of Russian disinformation efforts. It arguably constitutes the first documented attempt to achieve foreign political interference on a wider scale through deepfakes (see also ibid.).

How dangerous are deepfakes for foreign interference to democracy? None of the above-mentioned operations using deepfake profile pictures attracted a substantial following (ibid; see also Stone, 2020b). Moreover, the respective disinformation campaigns would likely also have been conducted without deepfakes. E.g. the IRA has long used fake social media accounts to exploit political tension (Stone, 2020b); and deepfakes were part of much larger disinformation campaigns (Paterson & Hanley, 2020: 443). Similarly, the deepfake of the Ukrainian president was most probably part of a Russian disinformation campaign. Besides, it proved ineffective as it was easily exposed. Thus, no deepfake foreign influence operation to date has had a decisive destabilizing impact on democracy.

However, deepfakes’ future use for foreign interference is uncertain (Nimmo et al., 2020: 16): Deepfake *images* cannot be traced or recognized—unlike real portraits. Yet, according to analytics firm Graphika, their use is also a paradox, as they introduce new cues to unmask fakery. Foreign actors may currently thus simply be trying out the technology (Bastian, 2020). Also, discovery may be part of the rationale when it comes to election manipulation, as it further undermines the domestic public’s trust in elections (Paterson & Hanley, 2020: 443). Concerning deepfake *videos*, however, the Zelensky deepfake’s amateurism surprised observers, as a truly convincing deepfake video was “only a few hours of work away” (Bastian, 2022, own translation) and would arguably have had a greater impact. The deepfake also confirms fears by observers such as the Estonian intelligence service and the FBI predicting an increased use of deepfakes by foreign actors (in particular Russia) against Western democracies (Välisluureamet, 2021: 66; FBI, 2021) and respective warnings by Ukrainian authorities in the context of the war in 2022 (Bastian, 2022).

More convincing deepfakes for foreign interference could undermine certain groups’ inclusion in democratic processes if they “flood communicative forums and drown out opportunities for individuals to contribute or encounter diverse views” (Tenove, 2020: 529). In my opinion, their scale would need to increase significantly to have this effect.^28^ Ultimately, foreign interference aims to prevent empowered inclusion in the sense of the overall self-determination of a targeted nations’ citizenry. As Tenove (2020: 522) puts it, “it undermines national security and – in the international context – sovereignty”.^29^ The Zelensky deepfake exemplifies this as it aimed to change the course of an international war and, ultimately, to end Ukrainian sovereignty. To date, however, deepfake-based interference has not yet succeeded in threatening a whole demoi’s empowered inclusion.

Currently, such deepfakes mainly interfere with collective agenda and will formation. Some operations publish content favourable to their country of origin. Primarily, however, they aim to destabilize and polarize the target country (here the USA) by spreading divisive, highly partisan content and conspiracy ideologies. This may reduce the willingness of certain citizens who believe such content to rationally engage with and accommodate citizens with opposing views and thus harms epistemic quality—impeding deliberation.

Lastly, the perception that foreign actors interfered with domestic elections—if widespread—reduces trust in elections and thus the legitimacy of collective decision-making. Again, this highlights how deepfakes cause informational and, by extension, societal trust decay, thereby harming democracy. In the case of the 2020 US elections, however, both fears of *domestic* deepfakes and domestic claims that the elections were rigged arguably created more havoc than deepfake-based foreign interference, which was limited. Deepfakes’ future use for foreign interference operations and thus their respective impact on democracy remains uncertain.

### Liar’s Dividend

Deepfakes used for election manipulation, attacking political opponents, and foreign interference all contribute to informational and societal trust decay. This trust decay in turn enables the “liar’s dividend”, which is a central issue in discussions about deepfakes’ impact on democracy. I define the liar’s dividend as the opportunity for individuals criticized for certain statements or actions to simply deny the truthfulness of incriminating evidence by referencing the existence of deepfakes (see Chesney & Citron, 2019: 1785). The liar’s dividend can also be invoked by criticized individuals’ supporters, who can (create) doubt (about) certain acts that contradict their worldviews or are otherwise undesirable. This is enabled by deepfakes’ mere existence, use, and the associated trust decay. The term was coined by Chesney and Citron (2019: 1785) in their seminal contribution on deepfakes and has since been widely taken up in both academia and the news media.

An early example surrounds a video of Gabonese President Ali Bongo. The President had not publicly appeared for months, fuelling rumours that he was ill or dead. When his 2018 traditional New Year’s address was broadcast, speculations about the video’s veracity caused public unrest and even culminated in a failed military coup. Digital forensics could later find no evidence of tampering (Ajder et al., 2019: 10), but the speculation alone was sufficient to fuel internal divisions and even violence. Besides, Malayan and Indian politicians accused of sex scandals have claimed that respective video evidence was faked (Blakkarly, 2019; Sudeep, 2021).^30^ In the USA, former President Donald Trump asserted as early as in 2016 that the “Access Hollywood” video was manipulated, in which he boasted about harassing women. More recently, Trump supporters, including nationalists and QAnon devotees, have doubted the veracity of videos showing Trump tested positively for the coronavirus, condemning the Capitol attack, and conceding to Joe Biden, and of public appearances by Biden (Beaumont, 2021; Chheda, 2021; MacDonald, 2020). Less than a month after George Floyd’s murder by a police officer in May 2020, a Republican US congressional candidate released a “report” claiming that the video of his death was a deepfake aimed to stir racial tensions (Sonnemaker, 2021). In the context of the military coup in Myanmar, the army and authorities have recently also doubted the authenticity of recordings documenting human rights violations (Gregory, 2021).

How does the liar’s dividend affect democracy? It impedes *empowered* inclusion when doubts about the misconduct of public officials, political candidates, or representatives prevent citizens (or relevant bodies) from holding perpetrators accountable, e.g. by deselection (Warren, 2017: 48) or prosecution. The liar’s dividend also further marginalizes repressed communities, whom “society is already less likely to believe” (Pfefferkorn, 2021). This is, e.g., the case concerning police violence against the black community in the USA (ibid.).^31^ In authoritarian regimes, it is even more likely that the liar’s dividend will prevent consequences even for widespread human rights violations. Journalists and human rights advocates already feel the respective burden of proof being shifted to them (Gregory, 2021).

The liar’s dividend also erodes the epistemic quality of deliberation. As Chambers (2021: 149) notes, partisan actors strategically exploit and exacerbate existing epistemic uncertainty by spreading “fake fake news”, i.e. attacking “‘real’ facts and/or fact-based journalism by the accusation of fake news”. The liar’s dividend thus undermines democratic discourse by contributing to the (on-going) erosion of epistemic quality. Ironically, this threat increases with growing public awareness and education about deepfakes (Chesney & Citron, 2019: 1785). Chesney and Citron (2019: 1786) fear that it creates more “space for authoritarianism”: when rational argument is weakened, “those who take a hegemonic position in the discourse and whose opinion is most prominent” gain power.

Trust decay plays a two-fold role here: the liar’s dividend is not only *enabled by* waning trust in the authenticity of empirical evidence and the news media, but it also *contributes* to further trust decay—both in empirical evidence and facts and in democratic institutions and fellow citizens. E.g. the increasing pressure on journalists and human rights organisations to prove claims about human rights violations shows that the liar’s dividend furthers doubt in and even resentment towards journalists and activist as crucial institutions of democratic oversight.

This is connected to a second consequence of the liar’s dividend, namely polarization. Polarization impedes deliberation since it reduces mutual respect and audiences’ “deliberative disposition to weigh reasons and proposals” (Mansbridge et al., 2012: 24). In the case, e.g., of Trump’s condemnation of the Capitol attack, deepfake theories circulating in right-wing fora fuelled conspiracy ideologies. When citizens are segregated into such “like-minded ‘niches’”, it prevents them from “hearing the other side and developing respect for people with whom they disagree” (ibid.: 21). Other instances such as doubts about George Floyd’s murder aggravated wide-spread societal divisions such as racial tensions, and the Gabonese case even illustrates deepfakes’ potential to incite violent conflict.

The liar’s dividend—building upon the trust decay caused by deepfakes—thus aggravates epistemic uncertainty and polarization (and thus, in turn, further trust decay). It undermines the quality of deliberation and collective agenda and will formation and, by extension, the legitimacy of collective decision-making.

### Weakening News Media and Journalism

The existence of deepfakes and the associated trust decay also create specific challenges for individual news outlets and news media in general (Bovenschulte, 2019: 1), and deepfakes exacerbate existing challenges for journalists concerning fact-checking (Diakopoulos & Johnson, 2019: 1; Chesney & Citron, 2019: 1784). To verify media, journalists increasingly need to rely on deepfake detection technologies, which are neither perfect nor readily available. This may prevent journalists “from rapidly reporting real, disturbing events” as they doubt the veracity of supporting evidence (ibid.). When journalists are duped by deepfakes, on the other hand, this entails a (further) loss of public trust (and epistemic quality).

A case in point is the above-mentioned deception of a US Jewish newspaper in 2020 into publishing an anti-Palestinian article by a deepfaked persona. Tellingly, the newspaper—which removed the content—admitted it had not pro-actively checked the author’s identity and had since improved its safeguards (Satter, 2020). Other news outlets refused to repeal the article—calling into question their trustworthiness. One editor also opined that the case might decrease outlet’s future willingness to publish unknown voices (ibid.).^32^

Journalists also self-report the challenge of fact-checking potential deepfakes and the lack of available tools (Lytvynenko in WITNESS, 2020b). Correspondingly, news organisations are joining efforts to develop deepfake detection tools. E.g. Agence France-Presse is cooperating with Google on its “Assembler” platform (Cohen, 2020), and German radio broadcaster “Deutsche Welle” is part of the respective research project “Digger” (Bundesregierung, 2019: 8). Such cooperation, as well as learning resulting from being “fooled” by deepfakes, may contribute to strengthening quality journalism.^33^

Notwithstanding, uncovering deepfakes remains costly and fault-prone, and journalists arguably regard deepfakes more as a (additional) challenge than an opportunity. In particular, deepfake technology has simplified the manipulation of videos, leading to their proliferation. The sheer volume of video material means that news outlets would need entire visual investigation teams to fact-check it (Lytvynenko in WITNESS, 2020b). This is often prohibitively costly—and clearly beyond the reach of average social media users or citizen journalists (ibid.).

Deepfakes thus already complicate journalists’ work and weaken trust in the media, and they are a growing challenge. This undermines collective agenda and will formation (and to a certain degree accountable representation and thus *empowered* inclusion), as the media play many important roles in deliberative systems. They are “watchdogs over power, representatives of citizens and communities, knowledge translators, educators of citizens, and public advocates” (Mansbridge et al., 2012: 20). As the “transmitter[s] of reliable and useful information”, news media are crucial for epistemic quality (ibid.). Also, they “greatly affect the tone of civility and respect among citizens” (ibid.: 21), in turn enhancing or undermining deliberation.

## Deepfake Hate Speech and Democracy

Deepfakes are not only—and not even primarily—used to spread disinformation. In fact, the term “deepfakes” was coined in 2017—and the technology first became known to a broader public—when an anonymous Reddit user posted pornographic face-swap videos and later published the respective code on GitHub (Schreiner, 2019). Pornographic deepfakes have since proliferated. According to visual threat intelligence company Deeptrace (now Sensity.AI), in 2019, 96% of all deepfake videos online were pornographic, and all of them depicted women (Ajder et al., 2019: i, 2). Recently, pornographic deepfakes of men have surfaced (e.g. Namboodiri, 2021), but they remain exceptions.

I argue that non-consensual pornographic deepfakes constitute hate speech and thus threaten democracy, even when they are not created with “political intentions”, i.e. to silence individual political opponents.^34^ There is no consistent definition of the term “hate speech”. Media scientist Sponholz (2018: 51) approaches it as “the deliberate and often intentional degradation of people through messages that call for, justify and/or trivialise violence based on a category (gender, phenotype, religion or sexual orientation)” (author’s translation). As such, hate speech is not restricted to speech acts, but also encompasses, e.g., image-based communication (ibid.: 57) and can be unintentional.

While pornographic deepfakes initially depicted celebrities, non-consensual fake porn of ordinary women, including revenge pornography, is now proliferating due to deepfakes’ increasing accessibility (Hao, 2021). E.g. the app “DeepNude” allows users to “undress” clothed images of any woman. It was only trained on female bodies and thus only works on them. After a surge in interest, the app was officially deleted, but the code is since circulating online (Ajder et al., 2019: 8). In 2020, a Telegram bot based thereupon allowed users to create more than 100,000 nude images of women. Many victims were minors (Vincent, 2020).

To a certain degree, this continues existing phenomena of non-consensual fake pornography, but AI has arguably amplified the threat as it “makes deep fakes look ‘real’ so that they correspond with our observed reality” (Maddocks, 2020: 5). Deepfake pornography can cause severe psychological harm to victims, including anxiety and depression. It can disadvantage them, e.g., in their professional life (Citron, 2019: 1926–1928). It also threatens victim’s equality and freedom by breaching their sexual privacy (ibid.: 1874, 1882). In some cases, women have even changed their names or ended their online presence as a reaction to non-consensual deepfake porn (Hao, 2021).

Since pornographic deepfakes overwhelmingly depict women, the issue is highly gendered (Chesney & Citron, 2019: 1773). It targets women as a societal group or “category”—a central feature of hate speech (Sponholz, 2018: 60). Deepfake porn decreases women’s life chances and can prevent them from participating actively in public life—both online and offline. As such, it is an instrument to “control women”—and other minorities (Faife in WITNESS, 2020b): As black activist Collins-Dexter (in ibid.) argues, deepfake porn also disproportionately targets people of colour, LGBTQ + and other marginalized communities, and is used to “fetishize, dehumanize, minimize and render invisible black men and women”. An example is racist deepfake porn shared in white supremacist circles.

Deepfake pornography predominantly harms the core democratic norm of empowered inclusion: Like other forms of hate speech (Sponholz, 2018: 59), it exacerbates existing discrimination through intimidation and denigration. It entails a “loss of power” by the depicted, and “thrive[s] on conventions that historically undermine women’s claims to truth” (Maddocks, 2020: 4–5). Members of marginalized groups are prevented from participating in public life and contributing to political decisions that affect them (see also Jankowicz, 2021) as they are not afforded “equal protections that enable [them] to use [their] empowerments”, i.e. “equal rights to vote, speak, [and] organize” (Warren, 2017: 44).

Unlike the other deepfakes analysed, most deepfake pornography does not aim to deceive viewers (Maddocks, 2020: 4). It thus does not contribute to epistemic uncertainty. Nonetheless, it harms collective agenda and will formation by reducing mutual respect (see Warren, 2017: 48). The legitimacy of collective decisions is also weakened, as some citizens cannot “consider their interests to have been fairly represented and considered” in the political process (ibid.).

## Conclusion and Outlook

My contribution grounds the debate about deepfakes in problem-oriented and deliberative democracy theory. I outlined how different uses of deepfake technology weaken core democratic functions and norms. To do so, I structured and assessed the dispersed body of literature on political deepfakes, which is often cursory and lacks an ethical or political science focus. I also integrated numerous recent media reports on deepfakes.

My analysis highlighted how deepfakes used for certain kinds of disinformation cause informational and societal trust decay and how this in turn enables the liar’s dividend and weakens news media. Deepfake disinformation impedes citizens’ empowered inclusion in political debates and decisions that affect them, e.g. by hampering efforts to hold political representatives accountable or further marginalizing certain societal groups such as women or ethnic minorities. Deepfakes also undermine collective agenda and will formation by threatening the epistemic quality of deliberation as well as citizens’ mutual empathy and respect. This culminates in a decreased legitimacy of collective decisions taken, which is additionally threatened by pervasive (but mostly speculative) fears of deepfake election manipulation, undermining trust in elections and their outcomes. I also highlighted the political importance of deepfake hate speech, in particular pornographic deepfakes. Such deepfakes weaken citizens’ mutual respect and their empowered inclusion.

My contribution is limited in several ways: Firstly, my inductive systematization of deepfake use cases is not the only conceivable categorization.^35^ Secondly, only literature in German and English was reviewed. The body of literature also included numerous non-peer reviewed media, think tank, company, and government reports.^36^ Thirdly, I draw on a limited number of contributions on democracy theory all originating in the Global North. Future contributions considering other theories of democracy, including those from the Global South, will prove fruitful to further stimulate the political (science) and philosophical debate on deepfakes.

I also paint a grim picture of deepfakes’ political impact by focusing exclusively on malicious uses. However, deepfakes also bear enormous potential for political education and debate. E.g. deepfake satire heightens public awareness of the technology (Klingenmaier, 2020), criticizes the powerful, and contributes to public debate. Other prosocial uses include political activism and public awareness campaigns, educational deepfakes, e.g., in museums or schools, and political art (Bieß & Pawelec, 2020). Such deepfakes can educate the public, e.g., on new technologies, or past historical events. In a recent contribution, I offer a first overview of such pro-democratic uses of deepfake technology grounded in problem-oriented and deliberative democracy theory (Pawelec, 2022). However, such deepfakes’ impact on democracy is not unambiguous. E.g., they too might be deceptive and manipulative. Further, theoretically grounded research on such applications is thus needed to paint a balanced picture of deepfakes’ impact on democracy.

Notwithstanding these limitations, the present contribution structures a large amount of dispersed literature on deepfakes and democracy. It also advances a more theoretically grounded analysis of deepfakes by explicating the democratic goods they threaten. This may also provide a basis for more expedient policy and societal responses—albeit considering that these policies’ impact on democracy (e.g. on free speech), in turn, must always be analysed critically (Tenove, 2020: 520).

 Responses to deepfakes are often part of broader efforts to regulate AI and curb disinformation and hate speech online (see van Huijstee et al., 2021: 37ff). However, specific deepfake governance efforts are also increasing. These include technical developments, such as those furthered by Facebook’s Deepfake Detection Challenge 2020; legal initiatives in South Korea, Britain, and the USA to introduce criminal offences concerning deepfake pornography (Hao, 2021); new platform policies on synthetic media (e.g., Facebook, 2021a; Roth & Achuthan, 2020); and the European Commission’s recent proposal for an “Artificial Intelligence Act” which specifically considers deepfakes (European Commission, 2021: 69). A deeper understanding of deepfakes’ normative threat can help evaluate and adapt such specific initiatives (as well as broader AI, disinformation, and hate speech policies) and craft future policy responses.

E.g. the European Commission plans to impose transparency obligations for most deepfakes in its “Artificial Intelligence Act” but exempts deepfakes subject to freedom of expression or the arts—while considering “appropriate safeguards for the rights and freedoms of third parties” (ibid.). Based on my analysis, this provision urgently needs specification. Exceptions from the transparency obligations must be delineated, since transparency increases the epistemic quality of deliberation, thus enhancing collective agenda and will formation. Also, my analysis shows that polarizing, racist, and pornographic deepfakes will continue to take their toll on democracy even when labelled as such, threatening empowered inclusion and the mutual respect necessary for deliberation. To take this into account, the Commission could augment its proposal, e.g., with measures to algorithmically deprioritize or even ban certain deepfakes.

Pornographic deepfakes, specifically, are often neglected in the discourse surrounding deepfakes and appropriate policy responses. Given their impact on affected individuals, this is unacceptable (Cole in WITNESS, 2020a; Maddocks, 2020: 2; Jankowicz, 2021). My analysis additionally highlights their grave political impact even when lacking obvious political intent. Efforts to protect women against deepfake pornography must thus be increased.^37^ This includes advancing existing legal initiatives and initiating respective discussions, e.g., in the EU. Considering deepfakes’ current proliferation, this is vital to guarantee women’s equal democratic rights and empowered inclusion.

## Data Availability

The work is based on a theoretical approach as well as a literature review of publicly accessible academic contributions, media reports, and internet publications (e.g., by think tanks, start-ups, and civil society). Table 1 in the Appendix lists all (types of) sources used to detail individual cases or instances of deepfake use analysed in the paper.

## Appendix

**Table 1:** (Types of) Sources used for the analysis of individual deepfake use cases^a^

**Deepfake disinformation and democracy**		
**Election manipulation**		
*Source*	*Type of source* ^b^	*Description/summary*
Parkin (2019)	Newspaper article (*The Guardian*)	“Politicians fear this like fire. The rise of the deepfake and the threat to democracy” Long-read news article which details uses of deepfake technology for political satire and disinformation. The article also offers an overview over the state of technology and recent advancements, quotes politicians’ and news medias’ fears about future use of deepfakes and discusses potential countermeasures such as detection technologies and public awareness.
Goel and Frenkel (2019)	Newspaper article (*The New York Times*)	“In India Election, False Posts and Hate Speech Flummox Facebook” Details proliferation of hate speech and disinformation (but not deepfakes) in Indian parliamentary election 2019.
Frum (2020)	Article in political magazine (*The Atlantic*)	“The Very Real Threat of Trump’s Deepfake” Former US-president Donald Trump retweeted a manipulated image of his political opponent Joe Biden pulling grimaces. The author argues Trump is “testing the boundaries” of posting fakery before the 2020 presidential elections.
Cole (2020)	Article in political magazine (*Vice*)	“For the Love of God, Not Everything Is a Deepfake” The author argues that the video Donald Trump posted of Joe Biden was not a deepfake and does not severely threaten democracy.
Papenfuss (2020)	Newspaper article (*Huffpost*)	“Fake Video! Trump Tweets Creepy ‘Independence Day’ Spoof Starring Him” Trump posted a deepfake video of himself starring in “Independence Day”. The article details the video’s content and popular reactions to it.
Romm and Stanley-Becker (2020)	Newspaper article *(The Washington Post)*	“Suspicious robocall campaign warning people to ‘stay home’ spooks voters nationwide” 10 million voters in Michigan received suspicious calls urging them to stay home and not vote in autumn 2020. The robocalls featured a computerized voice.
Collins and Zadrozny (2020)	News media article (*NBC News*)	“How a fake persona laid the groundwork for a Hunter Biden conspiracy deluge” A fake “intelligence” document about Joe Biden’s son Hunter Biden was published by a fake persona with a deepfake profile picture. It circulated among right-wing opponents of Joe Biden and was intended to discredit him.
Johnson (2020)	Article in online technology magazine (*Venture Beat*)^c^	“Twitter labels video shared by Trump aide as ‘manipulated media’” Details several instances of “cheapfakes” posted by supporters of Donald Trump.
**Attacking political opponents and suppressing the opposition**		
*Source*	*Type of source*	*Description/summary*
Satter (2020)	News agency report (*Reuters*)	“Deepfake used to attack activist couple shows new disinformation frontier” Several Jewish newspapers published articles by a fake persona with a deepfake profile picture. One article accused two Palestinian activists of sympathizing with terrorists. The article also discusses the targeted newspapers’ differing reactions and the case’s immediate and broader implications.
Benzaquen (2020)	Article in political magazine (+ *972 magazine*)^d^	“‘Leftists for Bibi’? Deepfake pro-Netanyahu propaganda exposed” A right-wing Israeli Facebook group posted several “confessions” by people who allegedly used to support left-wing policies and now support Benjamin Netanyahu. The fake personas used deepfake profile pictures. The article discusses the political context of these “confessions” and their reach.
Facebook (2021b)	Company report (*Facebook*)	“February 2021 Coordinated Inauthentic Behavior Report” Regular report by Facebook on “coordinated inauthentic behaviour” on its platform, i.e. attempts to manipulate public opinion using fake accounts, and Facebook’s countermeasures.
WITNESS (2020a)	Webtalk hosted by human rights organization (*WITNESS*)	“Boundary lines? Deepfakes weaponized against journalists and activists: Samantha Cole (Vice) and Nina Schick in conversation with Assia Boundaoui (MIT Co-Creation Studio), Deepfakery Webtalk series, Vol. 4.” Journalists and policy consultants discuss the targeted use of deepfakes as part of a series of webtalks on deepfakes organized by WITNESS, a US-American non-profit organisation specialised in the use of video and other technology to document human rights violations.
Jankowicz (2021)	Newspaper article (*The Washington Post*)	“Opinion: The threat from deepfakes isn’t hypothetical. Women feel it every day.” Opinion piece by a scholar studying disinformation campaigns stating that deepfakes are being weaponized by foreign actors against women to discourage their political participation and undermine democracy.
KrASIA (2021)	Online article by private digital media company (*KrAsia*)^e^	“Did Myanmar’s military deepfake a minister’s corruption confession?” The article reports that a video of a “confession” by a minister in Myanmar stating that he bribed Aung San Suu Kyi might be a deepfake.
**Foreign interference**		
*Source*	*Type of source*	*Description/summary*
Bastian (2020)	Article in online technology magazine (*Mixed*)^f^	“Deepfakes. China-Propaganda bei Facebook und Instagram” Reports on Facebook’s deletion of fake profiles on Facebook and Instagram spreading pro-Chinese propaganda. The profiles partially used deepfake profile pictures.
Stone (2020a)	Online article by private digital media company (*Cyberscoop*)^g^	“Chinese accounts blast Trump, with help from AI-generated pictures” Reports on the Chinese influence operation “Spamouflage dragon” using fake accounts and criticizing then-US-president Trump.
Vavra (2021)	Online article by private digital media company (*Cyberscoop*)	“FBI alert warns of Russian, Chinese use of deepfake content” Reports on an alert of the US Federal Bureau of Investigation to US private industry warning of the imminent future use of deepfakes for foreign interference. The alert also details several past foreign influence campaigns using deepfakes.
Stone (2020b)	Online article by private digital media company (*Cyberscoop*)	“Russia’s IRA used phony news accounts on Facebook to discuss QAnon, coronavirus” Reports on activities of Russian troll farms ahead of the US 2020 presidential elections. The Russian “Internet Research Agency” imitated independent news outlets with fake accounts to spread conspiracy theories and polarize the US public.
Graphika (2020)	Company report/analysis (*Graphika*)^h^	“Step into My Parler. Suspected Russian Operation Targeted Far-Right American Users on Platforms Including Gab and Parler, Resembled Recent IRA-Linked Operation that Targeted Progressives” A 36-page in-depth report by analytics firm Graphika analysing alleged Russian disinformation campaigns via a website posing as a right-wing news outlet, via fake Facebook accounts, and on networks such as Gab and Parler.
Graphika and DFR Lab (2019: 2–5)	Joint company and think tank report/analysis (*Graphika, DFR Lab*)^i^	“Operation #FFS: Fake Face Swarm. Facebook Takes Down Network Tied to Epoch Media Group That Used Mass AI-Generated Profiles” A 39-page joint report by analytics firm Graphika and the Atlantic Council’s Digital Forensics Research Lab on “Operation Fake Face Swarm”, an anti-Chinese, pro-Trump network of fake profiles on social media using deepfakes.
Bastian (2022)	Article in online technology magazine (*Mixed*)	“Möglicher Selenskyj-Deepfake: Miserabel und dennoch historisch” Reports on a deepfake video of the Ukrainian president urging Ukrainians to surrender to Russia. The low-quality video had little impact, but better quality deepfakes are within easy reach. The video was also nonetheless “historic” as the first use of deepfakes for foreign political interference on a wider scale.
**Liar’s dividend**		
*Source*	*Type of source*	*Description/summary*
Ajder et al. (2019)	Company report/analysis (*Deeptrace*)^j^	“The State of Deepfakes: Landscape, Threats, and Impact” An early, widely cited report by a cybersecurity company on the state of the art, increased commodification, and concrete uses of deepfakes for pornography and in the field of politics. The report is cited here to detail the case of accusations that the New Year’s address by the Gabonese President was a deepfake, which led to riots and a military coup.
Blakkarly (2019)	News media article (*SBS News*)	“A gay sex tape is threatening to end the political careers of two men in Malaysia” Reports on videos allegedly showing a Malayan minister involved in gay sex acts and the minister’s denial thereof and discusses the case’s implications for LGBT rights.
Sudeep (2021)	Newspaper article (*Deccan Herald*)	“Is Ramesh Jarkiholi sex video a ‘deepfake’?” Reports on a sex video showing an Indian state minister. The minister insisted the video was deepfaked, and the article discusses the likelihood thereof.
Beaumont (2021)	Newspaper article (*The Guardian*)	“Donald Trump fans cry betrayal as he rebukes Capitol violence” Donald Trump belatedly condemned the violence committed during the storming of the US Capitol. Conspiracy theorists including QAnon supporters believed the video was a deepfake.
MacDonald (2020)	Article on entertainment news website (*Inquisitr*)^k^	“Producers Speculate That Donald Trump’s Post-Coronavirus Video Is A Deepfake” Several producers publicly stated that the video Donald Trump tweeted concerning his coronavirus infection was a deepfake.
Chheda (2021)	News media article (*International Business Times*)	“Biden’s Head Disappears on Video, Fueling Speculation of Hologram Being Used for His Appearances” Fact-checks (and rejects) accusations that a video of Joe Biden was deepfaked to conceal the president’s allegedly decreasing mental health.
Sonnemaker (2021)	News media article (*Business Insider*)	“‘Liar's dividend’: The more we learn about deepfakes, the more dangerous they become” Discusses the spread of deepfakes and the associated liar’s dividend, highlighting the case of a Republican congressional candidate denying the veracity of the video showing George Floyd’s murder.
Gregory (2021)	Article in online technology magazine (*Wired*)^l^	“Authoritarian Regimes Could Exploit Cries of ‘Deepfake’. Opinion” Opinion piece by Sam Gregory, Program Director at WITNESS on the challenges deepfakes pose for the work of human rights activists.
Pfefferkorn (2021)	Think tank analysis (*Brookings*)	“The threat posed by deepfakes to marginalized communities” A scholar specialized on cybersecurity and cryptography argues that deepfakes will further undermine marginalized communities’ political standing and, e.g., cast doubt over the veracity of videos documenting police brutality.
**Weakening news media and journalism**		
*Source*	*Type of source*	*Description/summary*
Satter (2020)	News agency (*Reuters*)	See ‘Attacking political opponents and suppressing the opposition’.
WITNESS (2020b)	Webtalk hosted by human rights organization (*WITNESS*)	“Not funny anymore: Deepfakes, manipulated media, parody and mis/disinformation: Jane Lytvynenko (Buzzfeed News), Karen Hao (MIT Tech Review) and Brandi Collins-Dexter (Color of Change) in conversation with Corin Faife (WITNESS), Deepfakery Webtalk series, Vol. 2.” Journalists and scholars discuss the boundaries between parody and disinformation and what can be done against the latter, as part of a series of webtalks on deepfakes organized by WITNESS.
Cohen (2020)	Company report (*Jigsaw*)^m^	“Disinformation is more than fake news. By Jigsaw” Article by a subsidiary of Alphabet Inc. (Google) discussing Assembler, the company’s platform to allow journalists and face-checkers to detect manipulated images.
Bundesregierung (2019)	Government report (*German federal government*)	“Beschäftigung der Bundesregierung mit Deepfakes. Antwort der Bundesregierung auf die Kleine Anfrage der Abgeordneten Manuel Höferlin, Frank Sitta, Grigorios Aggelidis, weiterer Abgeordneter und der Fraktion der FDP -Drucksache 19/15210” Reply by the German federal government to questions by parliamentarians of the German Liberal party about the government’s response to deepfakes. Details, e.g., the governments understanding of (the problems posed by) deepfakes, the state bodies working on the issue, and existing and required legal regulation.
**Deepfake hate speech and democracy**		
*Source*	*Type of source*	*Description/summary*
Schreiner (2019)	Article in online technology magazine (*Mixed*)	“Geschichte der Deepfakes. So rasant geht es mit KI-Fakes voran” Continuously updated article which traces the technical development and spread of deepfake images and videos from the invention of generative adversarial networks (GANs) to current technical improvements.
Ajder et al. (2019)	Company report/analysis (*Deeptrace*)	See ‘Liar’s dividend’. The report includes an analysis of websites publishing deepfake porn, targets thereof, and a case study of the app DeepNude.
Namboodiri (2021)	Newspaper article (*Times of India*)	“Deepfake clips: ‘Sextortionists’ target celebs” Reports on the arrest of three men for blackmailing Indian businessmen, bureaucrats, and celebrities with deepfake sex videos.
Hao (2021)	Article in technology magazine (*MIT Technology Review*)	“Deepfake porn is ruining women’s lives. Now the law may finally ban it” Details the devastating impact of deepfake pornography on women, its proliferation (including via, e.g., DeepNude), and the inadequacy of victim’s chances to take action against it. The article also reports on current efforts to adapt legal frameworks, e.g. in the USA and UK.
Vincent (2020)	Article on online technology portal (*The Verge*)^n^	“Deepfake bots on Telegram make the work of creating fake nudes dangerously easy” Bots on the messaging app Telegram based on the DeepNude code allowed the creation of more than 100,000 fake nude images of women on demand.
WITNESS (2020b)	Webtalk hosted by human rights organization (*WITNESS*)	See ‘Weakening news media and journalism’.
Jankowicz (2021)	Newspaper article (*The Washington Post*)	See ‘Attacking political opponents and suppressing the opposition’.

^a^This table only includes the sources used to detail individual cases or instances of deepfake use, not, e.g., journal articles offering a broader overview of deepfakes’ (potential) impact (unless they also detail individual deepfake use cases). The sources are listed according to their order of citation in the paper. The table was compiled following a suggestion by an anonymous reviewer

^b^I do not differentiate here between newspaper articles published online and in print. All sources were accessed online

^c^The article links both to original sources (tweets and videos), and other news outlets, including CNN, to substantiate the information given

^d^The + 972 magazine describes itself as an “independent, online, nonprofit magazine run by a group of Palestinian and Israeli journalists” (see https://www.972mag.com/about/, accessed 27 April 2022). It is, e.g., supported by the German party-affiliated political foundation Heinrich-Böll-Stiftung

^e^KrAsia is self-reportedly a „digital media company reporting on the most promising technology-driven businesses and trends in the world's emerging markets” (see https://kr-asia.com/about-us, accessed 27 April 2022). The case described here was also taken up by other news media such as Wired (see https://www.wired.com/story/opinion-the-world-needs-deepfake-experts-to-stem-this-chaos/, accessed 27 April 2022)

^f^Mixed describes itself as Germany’s first and most-read online magazine about mixed reality and the future of computing (see https://mixed.de/ueber-mixed/, accessed 27 April 2022). The article links to original sources, including Facebook

^g^Cyberscoop is a “media brand” reporting on cybersecurity (see https://www.cyberscoop.com/contact/, accessed 27 April 2022). The instances of deepfake use reported in this and the subsequent articles on Cyberscoop were also reported on, e.g., by analytics company Graphika, the US Federal Bureau of Investigation, or news media such as the Washington Post. Following respective links from the Cyberscoop articles allowed triangulation to help ensure the validity of the information reported on

^h^Graphika is a private analytics firm. It analyses the evolution and manipulation of social networks (see https://graphika.com/our-story, accessed 27 April 2022). It has worked together, e.g., with Facebook to uncover and block foreign interference campaigns (see Bastian, 2020)

^i^The Atlantic Council is a US-American public policy think tank. Its Digital Forensics Research Lab studies disinformation, exposes human rights abuses and seeks to build digital resilience (see https://www.atlanticcouncil.org/programs/digital-forensic-research-lab/, accesses 27 April 2022)

^j^Deeptrace is now known as Sensity (AI). The Dutch company works on deep learning technologies to monitor and detect deepfakes (see https://medium.com/sensity/tagged/deepfakes, accessed 27 April 2022). Their 2019 report was widely cited in academia, e.g., by Etienne (2021); Gosse and Burkell (2020); and Ruiter (2021)

^k^The website links to the respective tweets, allowing a verification of the information given

^l^Wired is an online magazine reporting on “how technology is changing every aspect of our lives—from culture to business, science to design” (see https://www.wired.com/, accessed 28 April 2022). The article cited here is an opinion piece by a human rights activist frequently cited in the debate on deepfakes and democracy (see e.g., the WITNESS webtalks listed here)

^m^The article was published on the online platform Medium

^n^The Verge considers itself an “ambitious multimedia effort”. It is a subsidiary of the US company Vox Media (see https://www.theverge.com/about-the-verge, accessed 28 April 2022). The deepfake “ecosystem” this article details was uncovered by Sensity (see footnote j). It was also subject of numerous other media reports (triangulation), e.g., by the BBC (https://www.bbc.com/news/technology-54584127, accessed 28 April 2022) and MIT Technology Review (https://www.technologyreview.com/2020/10/20/1010789/ai-deepfake-bot-undresses-women-and-underage-girls/, accessed 28 April 2022)

## Footnotes

1 This definition is based on the academic and media discourse surrounding deepfakes. It is technically broad and includes fake images generated using generative adversarial networks (GANs) and so-called face-swap videos—the technologies originally associated with the term “deepfake”—but also synthetic audio tracks of human voices and videos manipulating facial or body movements of a target person according to input from an actor or based on a given audio track, so-called lip sync or puppeteering technologies. Such technologies are often deemed “deepfakes” and have comparable ethical and societal implications to the original technologies. The definition is also normatively broad: it questions the negative connotations and the expectations of truthfulness and deception associated with the term “deepfake”. It includes benign applications of the same technologies, which are often also deemed “deepfakes”—potentially to generate political or commercial attention. This allows a broader, more balanced assessment of the technology’s risks and potential.

2 Disinformation differs from misinformation, which is unintentionally false or misleading. Disinformation is also broader than “fake news”, as it includes blends of fabricated content and facts and various forms of misleading content not resembling “news”, including manipulated audio-visual media (HLEG, 2018: 10).

3 It is crucial to note here that many deepfakes are protected by the right to free speech. Free speech is essential for political plurality. It is a core democratic right of politically equal citizens (Warren, 2017: 44). Responses to deepfakes are thus subject to a dilemma concerning the limits of tolerance: How to weigh individuals’ right to free expression against the protection of other citizens’ dignity and equality or core democratic functions such as collective agenda and will formation (see, e.g. Heinze, 2016: 2–3; Tsesis, 2009: 497)? This contribution does not claim to solve this dilemma. It focuses only on the political *impact* of deepfakes (rather than deepfake countermeasures).

4 I thank an anonymous reviewer for highlighting the importance of context and intent here.

5 I focus on democracy in nation-states here, since deepfakes’ impact is mostly debated in relation, e.g. to national elections and foreign (non-)interference.

6 Similarly, a Web of Science search on 10 May 2021 for the terms “deep fake” and “deepfake” revealed 91 contributions, of which 51 were from computer sciences, 25 from the field of electrical and electronic engineering, and seven from the field of law. While eleven communications studies were found, only five political science/international relations and two ethics or philosophy contributions were included.

7 Also, not least due to the contribution’s pioneering nature, many deepfake use cases remain hypothetical and anticipatory (see Chesney & Citron, 2019: 1776), and scenarios are, e.g. based on past attacks by Russian hackers and authorities on the USA relying on very different technologies. While deepfake research is always a moving target, the greater range of existing deepfake use cases today allows an analysis drawing on a broader range of empirical data.

8 Other empirical contributions focus on narrower aspects of deepfakes’ political impact. Meneses (2021) analyses why deepfakes did not disrupt the US 2020 elections. Experimental research is also emerging. Dobber et al. (2020) study a microtargeted deepfakes’ impact on political attitudes and show that microtargeting can amplify deepfakes’ political impact. Experimental research by Barari et al. (2021, preprint), on the other hand, suggests that deepfakes defaming politicians are no more credible and effective than other forms of misinformation.

9 The above-mentioned chapter by the author on deepfakes’ positive potential for democracy (Pawelec, 2022) offers such an analysis—albeit not of the threats deepfakes pose to democracy.

10 Tenove (2020: 517) identifies three core “normative goods of democratic systems”, namely “self-determination, accountable representation, and public deliberation”. However, these goods are, as Tenove (2020: 518) himself states, inseparable. E.g. he argues that deliberation promotes democratic inclusion (ibid.: 528). However, inclusion is in turn crucial for self-determination of those affected by decisions and their ability to hold representatives accountable. Tenove’s identified core goods also operate at different levels: while self-determination is a normative good, representation and deliberation are arguably practices (Warren, 2017). Lastly, Tenove (2020: 518) emphasizes foreign non-interference to ensure self-determination but in my opinion neglects the importance of various domestic groups’ political inclusion for such self-determination—independent of foreign actors.

11 I thank an anonymous reviewer for highlighting the importance of trust for the issue at hand.

12 E.g., Habermas (2005: 288) identifies two arenas for political opinion and will formation: a wider public sphere, in which citizens debate political issues and form opinions and preferences, and smaller institutions such as parliaments responsible for decision-making. Mansbridge et al. (2012: 9) distinguish four arenas of deliberation, i.e. binding state decisions, “activities directly related to preparing these”, related “informal talk”, and “arenas of formal or informal talk related to decisions on issues of common concern that are not intended for binding decisions by the state”.

13 Warren simply deems this “trust” rather than societal trust. I thank an anonymous reviewer for the cited reference.

14 Due to its timely and “historic” (Bastian, 2022) nature, a later deepfake of the Ukrainian president in the context of the war in Ukraine (which surfaced during this paper’s review process) was also included in the analysis. Furthermore, anonymous reviewers suggested the consideration of three contributions, namely Paterson and Hanley (2020); Etienne (2021); and Ruiter (2021).

15 I thank an anonymous reviewer for highlighting the issue of varying types of sources and suggesting the addition of the named appendix.

16 Tenove (2020: 521) describes his approach as a form of “non-ideal” normative analysis, which operates “between abstract ideal models and mere empirical descriptivism”.

17 In other words, I attempt to mention all relevant examples for the respective use case as derived from the literature, based on my methodology of selecting this literature. This approach leads to an at times cautious assessment of deepfakes’ current (and potential) impact, including the judgement that deepfakes’ threat for democratic elections to date is low.

18 Chesney and Citron (2019: 1786) use the term “trust decay” in connection with deepfakes, but do not differentiate different types thereof.

19 This is not to make the positivist argument that there is one “objective” truth. Rather, following Habermas (1999), all truth claims are “fallible, corrigible, and redeemed in discourses”, i.e. truth claims are established, tested, and justified within deliberative processes (Chambers, 2021: 153). I thus regard functioning deliberative processes and the establishment of widely acceptable truth claims as mutually constitutive.

20 Conversely, Etienne (2021) argues that deepfakes do not threaten but rather increase online trust. His argument is three-fold: Firstly, deepfakes are simply a new tool for manipulation and disinformation. Secondly, they are not mainly used for disinformation but for deepfake porn, and thirdly, “we never trust a piece of information”, but rather the person spreading it, and political distrust existed long before deepfakes (Etienne, 2021: 559). Etienne then argues that deepfakes enhance online trust by heightening people’s critical awareness online (ibid: 560). I agree with Etienne on the continuity of deepfakes with other forms (and impacts) of manipulation and the overwhelmingly pornographic use of the technology. However, as I argue above, deepfakes enhance challenges associated with other forms of disinformation, and I show below that deepfake hate speech has a strong political component. I also disagree that deepfakes’ potential to sensitize people to potential video manipulation (and even that their potential pro-social uses, which Etienne does not mention) outweighs their political harms, and the present analysis serves to highlight this. Concerning this argument, Etienne also does not convincingly show why deepfakes—given their continuity with other forms of manipulation and misleading information—create unique opportunities for awareness creation and media competence. In my opinion, Etienne (ibid: 560–561) also places excessive responsibility (and expectations) on individual citizens to question the credibility of online information and search for alternative sources, and on individual journalists (and influencers) to verify information.

21 While the liar’s dividend is central to the discussion of deepfakes and democracy, it is (like a weakening of the media) *enabled* by the trust decay caused by the previously analysed deepfake use cases, and I will therefore discuss it subsequently.

22 I differentiate such election manipulation (and targeted attacks on opponents) from “propaganda”, i.e. coordinated campaigns by foreign state(-sponsored) agents.

23 For the 2020 US presidential elections, Meneses (2021: 9) argues that efforts by social media platforms to curb deepfakes, new laws, and greater social awareness also contributed to deepfakes’ lack of impact but that this was a combination of factors unique to the election.

24 Jankowicz (2021) draws connections between the attack on Ayyub and attacks on female politicians using fake porn, e.g. in Ukraine and the USA. She argues that “women feel” the threat of deepfakes every day. However, the other mentioned cases used “cheapfakes”, and to my knowledge, the targeted use of *deepfake* porn for political purposes is still rare.

25 The evidence is “inconclusive”. According to the NGO WITNESS, it might also be a forced confession read from a teleprompter (in KrASIA, 2021).

26 A contentious case in terms of categorization as foreign interference is “Operation Fake Face Swarm”, a network of over 900 Instagram and Facebook accounts and pages (of which dozens relied on deepfake profile pictures) that was deleted in December 2019. The network was most likely associated with the Epoch Media group, an international far-right media company supporting the religious Falun Gong movement. The multi-lingual network posted both content “consistently hostile toward China” and the Communist Party and pro-Trump coverage (Graphika & DFR Lab, 2019: 2–5).

27 Shortly thereafter, a deepfake video was published in which (fake) Russian President Putin proclaimed the end of the war. However, its creator immediately clarified that the video was faked. It was thus not intended to deceive but rather a political satire (Bastian, 2022).

28 Deepfakes created by malicious external actors could also prevent citizens from voting by spreading disinformation about election procedures or intimidating voters, e.g., through blackmail. To my knowledge, this scenario has not materialized so far (it is conceivable that cases of blackmail have not been made public).

29 In a globalized world, external actors have legitimate stakes in political processes abroad—within “justifiable limits”, including when their influence threatens citizens’ empowerment (Tenove, 2020: 522). Tenove argues such disinformation threatens the “normative good of self-determination” (ibid.). I argue that this can be subsumed under the wider democratic function of empowered inclusion.

30 The Malayan politician was “accused” of (consensual) same-sex activities, which are illegal in Malaysia (Blakkarly, 2019); I categorize this as an incident of the liar’s dividend since he and his supporters attempted to deflect blame by suggesting that the video was deepfaked (no evidence thereof was found). This is no judgment of the events (allegedly) depicted.

31 Notwithstanding, George Floyd’s murderer was found guilty and convicted in April 2021.

32 This case is also connected to a wider incident of 46 pro-Israeli news outlets publishing articles by 19 non-existent journalists from July 2019 to July 2020 (Middle East Monitor, 2020). However, I could find no reference of further deepfake use.

33 I thank an anonymous reviewer for this notion.

34 Non-pornographic deepfakes denigrating specific societal groups are also conceivable. However, I could find no evidence of such deepfakes so far.

35 E.g., it is conceivable to group cases of the targeted use of deepfake pornography to silence political opponents as deepfake hate speech.

36 However, I attempted to ensure the validity of the given information and a high degree of transparency concerning the types of sources employed (see the “Sect. 3” and Table 1 in the Appendix).

37 Creators of non-consensual deepfake porn often argue that it is protected by free speech (Burkell & Gosse, 2019: 8), which, as shown, is crucial for political equality and empowered inclusion. However, I believe victims’ well-being and dignity is of greater normative value than other individuals’ rights to denigrate fellow citizens using pornographic deepfakes (not least because this cannot be considered a meaningful expression of political opinion). Besides, by threatening large groups’ political participation, deepfake pornography threatens plurality itself.
